# Influences of adverse childhood experiences and weekly pregnancy stress on postpartum mental health symptoms: a machine learning examination

**DOI:** 10.1007/s00737-026-01715-0

**Published:** 2026-05-09

**Authors:** Amy K. Nuttall, G. Anne Bogat, Kevin J. Grimm, Maria Muzik, Joseph S. Lonstein, Cecilia Martinez-Torteya, Alytia A. Levendosky

**Affiliations:** 1 Department of Human Development and Family Studies, Michigan State University, East Lansing, MI USA; 2 Clinical Science Program, Department of Psychology, Michigan State University, East Lansing, MI USA; 3 Quantitative Methods, Department of Psychology, Arizona State University, Tempe, AZ USA; 4 Departments of Psychiatry, Obstetrics & Gynecology, University of Michigan – Michigan Medicine, Ann Arbor, MI USA; 5 Behavioral Neuroscience Program, Department of Psychology, Michigan State University, East Lansing, MI USA; 6 Department of Psychiatry, University of Michigan – Michigan Medicine, Ann Arbor, MI USA

**Keywords:** Pregnancy stress, Postpartum mental health, Depressive symptoms, Anxiety symptoms, Adverse Childhood Experiences, Machine learning

## Abstract

**Purpose:**

The goal of this study was to understand how the timing, level, and fluctuation of pregnancy stress affected women’s postpartum mental health while also considering their experiences of early life stress (adverse childhood events; ACEs). We hypothesized that overall stress level, as well as stress during particular pregnancy weeks, would influence postpartum mental health. We further hypothesized that fluctuations in weekly perceived stress levels across pregnancy would influence postpartum mental health among women who had experienced ≥ 4 ACEs.

**Methods:**

We collected information on ACEs and weekly stress data from our participants during pregnancy (15 weeks until parturition). Women’s depressive and anxiety symptoms were assessed at six-months postpartum. We employed a machine learning approach to explore the predictive utility of timing, level, and fluctuation of pregnancy stress on postpartum mental health symptoms.

**Results:**

For all women, higher average stress level across all weeks of pregnancy predicted greater postpartum depressive and anxiety symptoms. Additionally, women with a history of ≥ 4 ACEs were susceptible to stress levels in weeks 20 of pregnancy (depressive symptoms only) and 26 (depressive and anxiety symptoms). Lastly, greater variability in stress levels across pregnancy was also associated with greater depressive symptoms for the women with ≥ 4 ACES.

**Conclusion:**

In addition to overall stress level, timing of pregnancy stress and fluctuations in stress levels across pregnancy are associated with postpartum mental health for women with a history of substantial ACEs, with implications for focused mental health interventions during pregnancy for these women.

## Article Highlights

High stress across pregnancy predicted more postpartum depressive and anxiety symptoms.

For women with a history of a significant number of adverse childhood experiences, stress levels during week 20 of pregnancy were related to elevated levels of depressive symptoms postpartum.

For women with a history of a significant number of adverse childhood experiences, stress levels during week 26 of pregnancy were related to elevated levels of both depressive and anxiety symptoms postpartum.

For women with a history of a significant number of adverse childhood experiences, greater variability of levels of stress across pregnancy were related to elevated levels of depressive symptoms postpartum.

## Introduction

Numerous studies document high rates of maternal depression and anxiety during the postpartum period (Hahn-Holbrook et al. 2018). Indeed, ~ 10–20% of women have clinical levels of depression during the first year postpartum (Horowitz and Goodman 2005) and many more have high but clinically subthreshold levels (Woody et al. 2017). Postpartum anxiety is less studied, but rates range from 13 to 40% (Field 2018). Importantly, stressors during pregnancy are a significant predictor of mental health during the early postpartum period and beyond (Aris-Meijer et al. 2019; Ding et al. 2023; Agnafors et al. 2023).

Unfortunately, research has not yet determined which aspects of pregnancy stress–the timing, absolute levels, and/or fluctuation of stress across pregnancy–most influence postpartum mental health. Additionally, research has typically not distinguished the differential effects of early-life stress (e.g., childhood abuse) from pregnancy stress on postpartum mental health. The goals of the present study were to delineate which aspects of stress across pregnancy affected postpartum mental health and to consider the influence of early life stress on the impact. Pregnant women prospectively reported on their weekly perceived stress level from 15 weeks until parturition and retrospectively reported on adverse childhood events (ACEs). We assessed depressive and state anxiety symptoms six months postpartum, when women are at the highest risk for postpartum depression (O’Hara and McCabe 2013), and when many women experience sustained postpartum anxiety (Dennis et al. 2017).

A substantial body of research examines stress during pregnancy and its relation to postpartum mental health. Two reviews found that pregnancy stress predicted postpartum depression (Schalla and Stengel 2024; Yim et al. 2015). This was true when researchers measured specific stressors or multiple stressors. A meta-analysis concurred that pregnancy stress increased prevalence of postpartum depression and depressive symptoms, but at different rates depending on when the postpartum measurement occurred (Ding et al. 2023).

Unlike depression, few studies examine the effect of pregnancy stress on postpartum anxiety. Aris-Meijer et al. (2019) found that 3rd -trimester stressful life events were significantly related to women’s anxiety 5 months postpartum. DiPaolo et al. (2022) studied women’s objective and subjective hardship (proxies for stress) during pregnancy and both predicted anxiety two months postpartum. In a retrospective study where women completed questionnaires 2–9 months postpartum, more pregnancy stressors were related to greater postpartum anxiety (Farr et al. 2014).

Although it is well-established that pregnancy stress has a strong influence on maternal postpartum mental health, two significant methodological problems affect understanding the relations between these variables. First, research has not adequately examined stress multiple times across pregnancy to consider whether *fluctuations* in stress levels across pregnancy and *timing* of stress during pregnancy are important. To assess fluctuations and timing of stress, longitudinal, repeated assessments of pregnancy stress are required. Most studies measure pregnancy stress once (Aris-Meijer et al. 2019), implicitly assuming that stress levels are constant. However, several studies with multiple measurements of pregnancy stress indicate that stress levels fluctuate, although the findings are inconsistent For example, two studies that measured stress 3 times during pregnancy found that it decreased from early to late pregnancy (Asselmann et al. 2020; Cheng et al. 2021), whereas another study with 3 stress measurements conducted during the COVID-19 pandemic found elevated stress levels throughout pregnancy (Rabinowitz et al. 2023). Rallis et al. (2014) assessed women 6 times from weeks 16 to 36 and found that, on average, women experienced more stress symptoms at the beginning and end of pregnancy and less in the middle stage.

Frequent, repeated measurements of women’s stress levels throughout pregnancy also could allow for examining whether the *timing of stress* affects postpartum mental health. When assessments were taken 3 times during pregnancy, Scheyer and Urizar (2016) found that stress in each trimester was independently associated with depression at 3 months postpartum. Cheng et al. (2021) found mid-pregnancy stress (23–28 weeks) associated with postpartum depression, but other time periods when stress was measured (32–36 weeks and > 36 weeks) were not. Finally, Sun et al. (2024) collected stress, anxiety, and depression measures from women at < 22 weeks of pregnancy, only retaining women with mild anxiety in the study. These measures were also administered during the third trimester and 6 weeks postpartum. Stress at ≤ 22 weeks gestation was not related to postpartum depression or anxiety; however, stress during the third trimester was. Unfortunately, these three studies measured stress at different times during pregnancy, used different stress, depression, and anxiety measures, and collected outcomes at different timepoints postpartum. Even trimesters are not easily compared across studies as these are broad categories and, without more information, one cannot ascertain whether all participants *within* a given study were assessed at the same week during the trimester or whether assessment weeks *across* studies were similar. In addition, because these stress assessments encompass broad ranges of time, we cannot pinpoint specific *weeks* when women might be more vulnerable to stress’ effects.

In addition, individual women differ in their overall stress levels; thus, it is important to contextualize a woman’s weekly stress level against her average stress level. To our knowledge, only one study has done so. Racine and colleagues (2019) found that women with elevated stress above their own average at one timepoint were more likely to experience anxiety at the next timepoint above their own average level of anxiety. Unfortunately their statistical analysis did not allow conclusions about the impact of pregnancy stress timing on postpartum anxiety.

The second significant limitation in the literature is that few studies of pregnancy stress and its effect on postpartum mental health account for the effects of ACEs *in addition to* current pregnancy stressors. ACEs are important determinants of mental and physical health in adulthood (e.g., Hughes et al. 2017; Reid-Ellis et al. 2025). Importantly, women who experienced a significant number of ACEs are more affected by pregnancy stress (Ilori et al. 2025; Oh et al. 2016) and are more likely to have problematic mental health postpartum (e.g., Shin et al., 2021; Racine et al. 2021). Because many ACEs relate to the parenting one received as a child (abuse, neglect, household dysfunction), memories and emotions associated with these experiences may become activated during pregnancy and postpartum as women think about themselves as parents (Oosterman et al. 2019). However, the research methods of these studies did not determine the unique role of pregnancy stress from that of past stressors on postpartum mental health.

Fluctuations in pregnancy stress might be especially important for women who experienced substantial ACEs. These women may be more sensitized and reactive to proximal stressors and thus have more variability in perceived stress levels across pregnancy. This hypothesis corresponds with current conceptualizations of sensitivity for Reproductive Mood Disorders (Schweizer-Schubert et al. 2021). Some women, due to genetic makeup and epigenetic modification (e.g., after childhood trauma), have heightened sensitivity to fluctuations in reproductive and stress-related steroids. This heightened sensitivity to stress fluctuation may promote risk for reproduction-related affective disorders.

## The present study

We investigated how the level, fluctuation, and timing of pregnancy stress from 15 weeks to parturition affected women’s postpartum mental health. We expected women’s mean level of stress across pregnancy to predict both postpartum depressive and anxiety symptoms. We also hypothesized that stress during certain weeks (rather than levels during broad trimesters) were important influences on postpartum mental health; however, we did not advance hypotheses about specific weeks. We further hypothesized that women who had experienced substantial ACEs were most susceptible to pregnancy stress. Specifically, that fluctuations in weekly stress across pregnancy would influence postpartum mental health among women who had experienced ≥ 4 ACEs. We did not advance differential hypotheses for either depressive symptoms or anxiety symptoms. Given the large number of predictors (29 pregnancy stress variables), we employed a machine learning exploratory approach to assess the predictive utility of each of these on postpartum mental health by ACEs status.

## Methods

### Participants

Participants were enrolled in a longitudinal study investigating the effects of pregnancy stress on later maternal and child outcomes (blinded). Women were recruited as early as 15 weeks in their pregnancies (*M* = 17.64 weeks, *SD* = 2.54) through social media, flyers, community organizations, Ob/Gyn clinics, and an Ob/Gyn Perinatal Registry. Enrollment criteria included: English fluency, ages 18–34 years, no medical issues impairing cortisol production, in a heterosexual relationship during the current pregnancy, and < 20 weeks pregnant with a singleton. We oversampled for intimate partner violence (IPV), because IPV is the most common traumatic stressor for pregnant women (Mendez-Figueroa et al. 2013). Women not experiencing IPV had to have ≥ 2 stressors strongly associated with IPV (e.g., low income, food insecurity) so that the sample of women experiencing IPV would be similar to those without IPV.

396 women completed the weekly pregnancy stress surveys. Of these, 265 completed mental health measures at six months postpartum. See Table 1 for demographics.

**Table 1:** Participant Demographics (*N* = 265)

Demographic	M	SD
Age (in years)	27.32	4.35
Monthly family income (in US dollars)	$2,830	$2,413
	**N**	**%**
Race		
Native American	2	0.8
Asian	6	2.3
Black	99	37.4
Multi-Racial	19	7.2
White	131	49.4
Latina, Hispanic, Chicana	1	0.4
Other	2	0.8
Education		
High school or less	147	55.47
Some college or more	117	44.2
Cohabitation Status		
Not Living with Partner	70	26.4
Living with Partner	168	63.4

### Procedures

During the first pregnancy assessment, women were administered the ACEs instrument. Following the first assessment, and throughout their pregnancies, women were sent weekly online stress questions by text or email. At 6 months postpartum, women completed the depressive and anxiety symptoms scales.

### Measures

#### Adverse childhood experiences

(ACEs; Felitti et al. 1998) is a 10-item scale measuring stressful/traumatic experiences before age 18. We divided our sample into two groups: women with *≥* 4 ACEs and those experiencing fewer. This grouping employed a widely-used cut-point (Felitti 2002) with predictive validity identifying mental health problems (Hughes et al. 2017).

#### Weekly stress

From enrollment until parturition, women were sent a weekly questionnaire that included a question to indicate, on a 0–6 scale (no stress to high stress), how stressed they felt during the past week. They were given a week to answer this question. Research indicates the validity of a single item to assess stress (Elo et al. 2003). Based on women’s responses to the weekly stress question, we first calculated women’s average stress level (one variable representing each woman’s mean across weeks of pregnancy). Second, we calculated each woman’s fluctuation in stress levels across pregnancy (one variable representing each woman’s standard deviation across weeks of pregnancy). Third, we calculated a timing variable for each week which was a weekly deviation stress score; that is, the difference between each participant’s weekly stress score and their mean stress score (a person-mean centered approach; Wang and Maxwell 2015). We expected a woman’s own mean level of reported stress was a better comparison for her other weeks than a between-persons approach that would suggest that all women rate their stress levels the same.

#### Edinburgh postnatal depression scale

(EPDS; Cox et al. 1987) was administered at 6 months postpartum. It is a widely used measure of postpartum depressive symptoms. Total scores were calculated from 10 items rated on a 4-point scale (α = 0.89).

#### State trait anxiety inventory (STAI) – state version

Spielberger et al. (1983) was administered at 6 months postpartum. It is a widely used measure of state anxiety validated for postpartum women (Meades and Ayers 2011). Total scores were calculated from 20 items answered on a 4-point scale (α = 0.94).

### Analytic approach

We accounted for missingness and conducted machine learning variable selection using a three-step approach. First, we used multiple imputation (Rubin 1987) to prepare 21 datasets for analysis with no missingness using M*plus* (Muthén & Muthén, 1998–2017). The ordered nature of the weekly stress variables was recognized during imputation using an ordered logistic regression model. Within each dataset, the 29 stress variables were calculated (over time mean and standard deviation, stress deviation scores for weeks 15–41) and merged with postpartum mental health data; participants missing mental health data were dropped. Second, we performed the machine learning procedure in each of these imputed datasets. We used Adaptive least absolute shrinkage and selection operator (LASSO) regression (Tibshirani 1996; Zou 2006), a variable selection technique that penalizes regression estimates and accounts for the different scales of explanatory variables through the use of adaptive weights. Regression estimates are found by minimizing the loss function:

$$\:{\sum\:}_{i=1}^{N}{\left({y}_{i}-{\widehat{y}}_{i}\right)}^{2}+\lambda\:{\sum\:}_{j=1}^{p}\left|{\beta\:}_{j}\right|$$

where the first term is the residual sum of squares (typical loss function in ordinary least squares regression) and the second term is the penalty function, where $\:\lambda\:$, the tuning parameter, is multiplied by the sum of the absolute values of the regression coefficients ($\:{\beta\:}_{j}$). Different values of the tuning parameter $\:\lambda\:$ are specified and an optimal value is determined through internal cross-validation (e.g., *k*-fold cross-validation) of the mean squared error. As $\:\lambda\:$ increases, regression estimates shrink toward zero; when regression estimates shrink to zero, the explanatory variable is removed from the model. The glmnet R package was used with its built-in *k*-fold cross-validation function (cv.glmnet()) to conduct the Adaptive LASSO with *10*-fold cross-validation (Geisser 1975). This was done for the whole sample and again separately for women who experienced ≥ 4 (*n* = 108) and < 4 ACEs (*n* = 153). Third, we summarized results across the imputed datasets; see Table 2 note for details.

**Table 2:** Results across adaptive LASSO models

**Depressive Symptoms Outcome**																	
**Model**		**M**	**SD**	**15**	**16**	**17**	**18**	**19**	**20**	**21**	**22**	**23**	**24**	**25**	**26**	**27**	**28**
1. Full Sample	# of imputations	**21**	**15**	0	5	1	3	9	3	5	1	0	0	5	4	2	0
	Coefficient	**1.74**	**0.75**														
	Effect Size	**0.35**	**0.05**														
2. ACEs = 4 or >4	# of imputations	**19**	**12**	7	8	2	3	3	**12**	3	0	0	2	0	**21**	1	2
	Coefficient	**0.91**	**0.80**						**0.27**						**0.81**		
	Effect Size	**0.18**	**0.05**						**0.07**						**0.22**		
3. ACEs < 4	# of imputations	**21**	1	4	4	2	2	7	2	1	0	0	4	5	0	2	0
	Coefficient	**1.93**															
	Effect Size	**0.39**															
**Model**		**M**	**SD**	**29**	**30**	**31**	**32**	**33**	**34**	**35**	**36**	**37**	**38**	**39**	**40**	**41**	
1. Full Sample	# of imputations	**21**	**15**	3	0	4	0	1	0	0	3	1	3	4	6	1	
	Coefficient	**1.74**	**0.75**														
	Effect Size	**0.35**	**0.05**														
2. ACEs = 4 or >4	# of imputations	**19**	**12**	1	0	0	2	0	0	1	1	1	0	2	0	3	
	Coefficient	**0.91**	**0.80**														
	Effect Size	**0.18**	**0.05**														
3. ACEs < 4	# of imputations	**21**	1	0	1	1	1	0	2	2	0	0	1	3	1	1	
	Coefficient	**1.93**															
	Effect Size	**0.39**															
**Anxiety Symptoms Outcome**																	
**Model**		**M**	**SD**	**15**	**16**	**17**	**18**	**19**	**20**	**21**	**22**	**23**	**24**	**25**	**26**	**27**	**28**
1. Full Sample	# of imputations	**21**	0	2	1	1	0	1	1	0	0	1	1	2	1	6	0
	Coefficient	**4.02**															
	Effect Size	**0.37**															
2. ACEs = 4 or >4	# of imputations	**20**	2	3	6	4	2	0	5	3	1	3	3	1	**21**	3	1
	Coefficient	**1.93**													**1.68**		
	Effect Size	**0.16**													**0.20**		
3. ACEs < 4	# of imputations	**21**	0	4	1	1	0	0	0	1	0	0	4	0	1	4	0
	Coefficient	**4.45**															
	Effect Size	**0.42**															
		**M**	**SD**	**29**	**30**	**31**	**32**	**33**	**34**	**35**	**36**	**37**	**38**	**39**	**40**	**41**	
1. Full Sample	# of imputations	**21**	0	0	0	0	0	0	0	0	0	0	1	0	1	2	
	Coefficient	**4.02**															
	Effect Size	**0.37**															
2. ACEs = 4 or >4	# of imputations	**20**	2	6	5	7	5	5	3	6	0	3	1	1	2	4	
	Coefficient	**1.93**															
	Effect Size	**0.16**															
3. ACEs < 4	# of imputations	**21**	0	0	1	0	1	3	0	1	0	0	1	0	0	1	
	Coefficient	**4.45**															
	Effect Size	**0.42**															

Predictor variables are: M = mean stress level across pregnancy; SD = standard deviation across pregnancy; 15–41 = weekly stress deviation scores (the difference between each participant’s raw weekly stress score and their mean stress level across pregnancy) for weeks 15–41 of pregnancy. # of imputations = the number of times the variable was selected by machine learning across the 21 imputed datasets; variables selected in 10 or more of the 21 imputed datasets were retained and are bolded. Coefficient = the unstandardized regression coefficient averaged over the 21 imputed datasets; the unstandardized regression coefficient was calculated and reported if the variable was retained in 10 or more of the 21 imputed datasets. Effect size = the corresponding standardized regression coefficient calculated by multiplying unstandardized coefficients by the ratio of the standard deviation of the associated predictor and the standard deviation of the outcome

## Results

Across pregnancy, women’s mean stress was 3.01 (*SD* = 1.20; range 0–5.63.63) and their weekly stress standard deviation was 1.41 (*SD* = 0.37; range 0–2.63.63). Results are in Table 2.

### Depressive symptoms

For all women, the average stress level across all weeks predicted depressive symptoms, with more stress associated with more depressive symptoms. For women with ≥ 4 ACEs: (1) more variability in their stress levels was associated with more depressive symptoms; and (2) higher stress levels in weeks 20 and 26 of pregnancy, compared to their average, were associated with more depressive symptoms.

### Anxiety symptoms

For all women, their average stress level across all weeks predicted anxiety, with more stress predicting more anxiety. Women with ≥ 4 ACEs were susceptible to more stress in week 26, such that more stress, compared to their average, was associated with more anxiety symptoms.

## Discussion

Our innovative research collected weekly stress ratings from week 15 of pregnancy until childbirth. This allowed us to assess women’s average stress level across pregnancy, determine weekly variability in stress, and compare each woman’s weekly stress level to her average stress level. We found that women’s postpartum symptoms were predicted by individual differences in women’s stress levels across pregnancy (average stress level, magnitude of fluctuation) as well as differences in the timing of their stress across pregnancy. The patterns of findings for both depressive and anxiety symptoms differed based on whether or not women experienced ≥ 4 ACEs.

For all women, the average stress level throughout pregnancy was associated with both postpartum anxiety and depressive symptoms. This finding confirms and extends prior literature. Moreover, our results for depressive symptoms relate to findings in other literature about the importance of fluctuations. For example, the degree to which individuals fluctuate from their “core affect” is linked to well-being and psychopathology over and above mean levels of emotions (Houben et al. 2015; Kuppens et al. 2010).

Rather than relying on a one-time measurement of pregnancy stress, which assumes stress is constant across pregnancy, our weekly measurements allowed for more accurate representations of women’s mean stress levels over time. Moreover, our frequent measurements revealed two ways in which pregnancy stress was associated with postpartum mental health among women with *≥* 4 ACEs. First, for these women, fluctuation in perceived stress was associated with postpartum depressive symptoms, but not anxiety. The Contrast Avoidance Model of Worry (Newman and Llera 2011) suggests that individuals with generalized anxiety disorder use worry to prolong a negative emotional state to avoid unexpected negative emotional shifts. This may explain why fluctuations in stress were not associated with anxiety symptoms.

Second, only for those women experiencing ≥ 4 ACEs, increased stress in weeks 20 and 26 was associated with postpartum depressive symptoms and week 26 was associated with anxiety symptoms. Our findings for depressive symptoms correspond with Cheng et al. (2021) who also found mid-pregnancy stress (but not other weeks) associated with postpartum depression. At this time, the realities of giving birth and parenting may become more salient [woman feel fetal movements (de Vries and Fong 2006), learn their fetus’ sex (Harrington et al. 1996), and the fetus becomes viable *ex utero* with medical intervention (Ecker et al. 2016)]. However, our findings were specific to women with ≥ 4 ACEs, suggesting that these realities may evoke fears and worries related to their own negative childhood experiences and contribute to their poor mental health. Interestingly, for women with ≥ 4 ACEs, week 20 predicted depressive symptoms, but not anxiety. Furthermore, the weeks between weeks 20 and 26 did not predict depressive symptoms. Given the data-driven nature of machine learning, future work examining the influence of stress during this period will help determine whether the specific weeks we identified or the entire span of weeks between them critically influence mental health.

The normative physiological changes across pregnancy help prepare women neurobiologically for the complex role of parenting, but these hormones also render some women more vulnerable to mental health problems (Barba-Müller et al. 2019; Pawluski et al. 2021), especially in the context of pregnancy stress (La Marca-Ghaemmaghami et al. 2021; Peterson et al. 2020) and early-life stress (Moog et al. 2016; Swales et al. 2018). This disruption likely contributes to mental health problems during pregnancy (Castro-Quintas et al. 2024) and postpartum (Seth et al. 2016; Pawluski et al. 2024; Glynn and Sandman 2014). Pregnant women with more ACEs and trauma-induced affective reactivity may have heightened acute stress responses during pregnancy, rendering them most vulnerable for postpartum affective disorders (Schweizer-Schubert et al. 2021).

Our findings are inconsistent with limited extant research using multiple, but few, assessments of pregnancy stress as it relates to postpartum mental health, none of which examined ACEs. One study found that stress during each trimester related to postpartum depression (Scheyer and Urizar 2016), and another found only 3rd-trimester stress was related to postpartum anxiety (Sun et al. 2024). Our findings most closely match those of Cheng et al. (2021) who found that only stress during weeks 23–28 was associated with postpartum depression. Discrepant findings in the timing literature are, in part, likely explained by these studies having assessed absolute levels of stress at one timepoint rather than accounting for a woman’s average stress level across pregnancy as done herein.

Furthermore, Cheng et al.’s findings relate to their entire sample of low-risk women with unknown childhood experiences, whereas we only detected this association among women with ≥ 4 ACEs. Examining results in the full sample, rather than considering ACEs, would have led us to mis-conclude that fluctuating stress levels across pregnancy were associated with depressive symptoms for all women rather than just those with ≥ 4 ACEs. With regard to postpartum anxiety, examining results in the full sample rather than considering ACEs would have obscured our result that women with ≥ 4 ACEs were susceptible to increased stress in pregnancy week 26.

The strengths of our research include studying a large sample of vulnerable women with a range of stressors across pregnancy, as well as examining differences between two sub-groups using the well-established ≥ 4 ACEs cutoff. Our unique weekly stress assessment allowed us to examine whether timing of stress mattered for postpartum mental health. Furthermore, we contextualized stress by calculating each woman’s weekly stress as the deviation from her own average across pregnancy. Finally, our use of machine learning allowed us to include more predictors in the model than traditional regression models.

The study also has several limitations. First, we did not assess subgroups based on clinical diagnoses of depression or anxiety. Second, we only measured mental health at 6 months postpartum. We dropped women missing outcome data due to limitations of our machine learning approach. Finally, we do not have any pregnancy stress data before 15 weeks and not assessing earlier stress may have affected our results.

In summary, our research emphasizes the importance of measuring stress multiple times throughout pregnancy to understand its nuanced influence on postpartum mental health. Our findings also underscore the importance of assessing ACEs to understand which women are most affected by pregnancy stress. Pinpointing the weeks when stress is most harmful to postpartum mental health could lead to important interventions *during pregnancy* to prevent serious outcomes for the most vulnerable women.
